# Antiferromagnetic skyrmion-based high speed diode†

**DOI:** 10.1039/d2na00748g

**Published:** 2022-12-15

**Authors:** Namita Bindal, Ravish Kumar Raj, Brajesh Kumar Kaushik

**Affiliations:** a Department of Electronics and Communication Engineering, Indian Institute of Technology Roorkee Uttarakhand India 247667 namita@ec.iitr.ac.in ravish_kr@ece.iitr.ac.in bkk23fec@iitr.ac.in

## Abstract

Antiferromagnetic (AFM) skyrmions are favored over ferromagnetic (FM) skyrmions as they can be driven parallel to in-plane driving currents and eventually prevent the annihilation at the edges of nanotrack. In this study, an AFM skyrmion-based diode is proposed to realize the one-way skyrmion motion that is crucial for data processing in nanoelectronic and spintronic devices. The skyrmion transport is controlled by exploiting the staircase notch region in the middle of the nanotrack. By virtue of this, the micromagnetic interaction energy between the skyrmion and the notch edges generates a potential gradient that further gives rise to repulsive forces on the skyrmion. The resultant of the forces from the driving current and edge repulsions make the skyrmion move along the notch region to overcome the device window and reach the detection region. The notch is designed in such a way that it prevents the movement of the skyrmion in the reverse direction, thereby achieving diode functionality. The proposed device offers processing speed in the order of 10^3^ m s^−1^, hence paving the way for the development of energy-efficient and high-speed devices in antiferromagnetic spintronics.

## Abstract 2

An AFM skyrmion based diode is designed using a staircase notch region at the middle of the nanotrack. The notch region induces the change in potential energy and acts as a barrier, thus allowing the unidirectional motion of the skyrmion.

## Graphical abstract

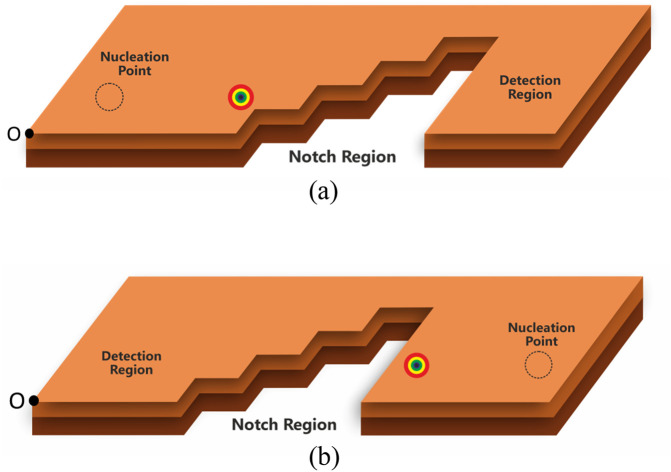

## 1 Introduction

Electrical diodes, operated under the effect of unidirectional current flux density, are the fundamental components of modern computation, communication, sensing, and artificial intelligence (AI) applications, enabling major advances in the fields of science and applied technology.^1,2^ Owing to the unidirectional operation of the electrical diode, considerable interest has been generated in various physical disciplines, including acoustics,^3^ microfluidics,^4^ photonics,^5^ thermodynamics,^6^ and micromagnetism.^7^ Magnetic skyrmions, a non-linear nanoscale spin texture, found in chiral magnets with broken inversion symmetry, can be viewed as viable information carriers due to their particle-like nature benefitting from unidirectional transport for diode applications.^8,9^ The unique properties of skyrmions, such as topological stability, nanoscale size, and ultra-low driving current density, make them ideal candidates for spintronic applications.^10,11^ Hence, skyrmions are predicted to bring in a new paradigm for spintronic devices.

With an extensive study of the nucleation, dynamics, and detection of FM skyrmions, numerous devices such as racetrack memories,^12,13^ transistors,^14^ logic gates,^15^ oscillators,^16^ diodes,^17^ neurons,^18^ and synaptic devices^19^ have been designed. Moreover, ferromagnetic (FM) skyrmion experiences.

Magnus force and hence deviate from the driving current direction and may be annihilated at device edges.^20^ Hence, the skyrmion Hall effect (SkHE) poses a challenge for realistic applications that require a straight motion of a skyrmion along the direction of the applied current.^21,22^ However, antiferromagnetic (AFM) skyrmions follow a straight trajectory in the current direction, which is very promising for next-generation spintronic applications.^23–25^ AFM skyrmions have two sublattices, coupled by the inter-sublattice exchange interaction with no net magnetic moment, thereby vanishing the dipolar fields and completely eliminating the SkHE. AFM skyrmions are thus not only mathematically attractive but also offer major advantages that make them viable alternatives to FM skyrmions. Owing to the insensitivity of AFM skyrmions towards external magnetic fields, they are more robust against magnetic field perturbations, thus improving the stability and reliability of AFM skyrmion-based devices.^26,27^ The velocity of these skyrmions is around tens of order in magnitude greater than that of FM skyrmions.^23^ Furthermore, AFM materials are more abundant in nature, which include metals comprising Mn-based alloys, insulators, and semiconductors.^28^ These intriguing features of AFM skyrmions have encouraged the recent development of AFM spintronics, which has opened up a path to the notions of magnetic devices that could eventually replace conventional FM counterparts.

Recently, several FM skyrmion based diodes have been proposed.^9,29–32^ Various methods have been employed to control skyrmion transport, such as high anisotropy regions, asymmetric structures, modified edges and the SkHE effect. To confine FM skyrmions in the middle of the nanotrack, high-Ku regions at the top and bottom are also incorporated, thereby consuming much higher energy.^29^ Hence, in this work, an AFM skyrmion is used to design a diode that does not intrinsically exhibit SkHE, thereby enabling ultra-low power consumption and ultra-high processing speed of the device. In the proposed device, a notch region in the middle of the AFM nanotrack is incorporated. The notch region acts as a barrier, and allows the unidirectional motion of the skyrmion, thereby achieving P–N junction diode functionality. The competition between the forces, due to the driving current (by the application of a spin–orbit-torque (SOT)) and edge repulsions from the notch, guide the skyrmion through the device window to reach the detection region while preventing its motion in the reverse direction. The combined effects of driving current and the dimensions of notch steps on skyrmion dynamics have been investigated using micromagnetic simulations. Our findings could have a significant impact on fundamental physics and could be valuable in the development of AFM skyrmion-based energy-efficient devices for various applications including transistors, directional couplers, high-speed magnetic field sensors, and many telecommunication devices.

## 2 Methodology

Micromagnetic simulations were carried out using MuMax3 software^33,34^ to calculate the space and time-dependent magnetization evolution in nanoscale magnets based on the quantization of the sample, where the cell quantization is less than the exchange length. An AFM film with two sublattices of reversely aligned spins having magnetic moments *m⃑*_1_(*r⃑*,*t*) and *m⃑*_2_(*r⃑*,*t*), |*m⃑*_1_| = |*m⃑*_2_| = *M*_s_/2 where *M*_s_ is the saturation magnetization was considered. The total magnetization was *m⃑*(*r⃑*,*t*) = *m⃑*_1_(*r⃑*,*t*) + *m⃑*_2_(*r⃑*,*t*) and the staggered magnetization was *l⃑*(*r⃑*,*t*) = *m⃑*_1_(*r⃑*,*t*) − *m⃑*_2_(*r⃑*,*t*). The former was used to describe the canting of magnetic moments, and the latter was used to obtain the unit Néel vector that is related to AFM order.^25^

The time-dependent magnetization dynamics were computed using the well-known Landau–Lifshitz–Gilbertz Slonczewski (LLGS) equation that is described as follows:^35^1
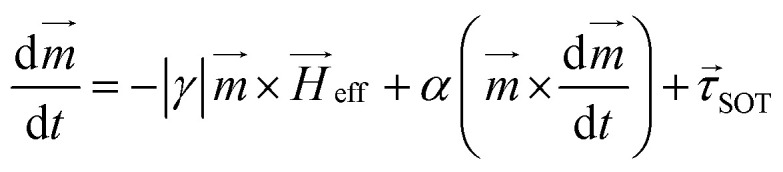
where *m⃑* = *m⃑*(*r⃑*,*t*) = *M⃑*(*r⃑*,*t*)/*M*_s_ represents the normalized magnetization. *α*, *γ*, and *M*_s_ are the damping constant, gyromagnetic ratio, and saturation magnetization, respectively.

*H⃑*_eff_ = −*δε*_AFM_/*μ*_0_*δm⃑* is the net field associated with various energies, such as magneto-crystalline anisotropy energy, exchange energy, and the Dzyaloshinskii–Moriya interaction (DMI) energy. The skyrmion is driven by the SOT mechanism that is given as follows:^36^

2 *τ⃗*_SOT_ = −*γm⃑* × (*m⃑* × *H*_0_*a⃑*_*y*_)

here, *H*_0_ = (ℏ*Jθ*_SHE_)/(2*eμ*_0_*t*_0_*M*_s_), where *ℏ*, *e*, *J*, *t*_0_, *θ*_SHE_, and *a*_*y*_ are Planck's constant, electron charge, charge current, thickness of the film, spin hall angle, and spin polarization unit vector, respectively. The following equation defines the overall AFM energy density as a function of *m⃑*:^35^

3 *ε*_AFM_ = *J*_ex_[(∇*m*_*x*_)^2^ + (∇*m*_*y*_)^2^ + (∇*m*_*z*_)^2^] − *K*_0_*m*_*z*_^2^ + *D*[*m*_*z*_(∇·*m⃑*) − (*m⃑*·∇)*m*_*z*_]

where the first, second, and third terms on the right hand side denote the micromagnetic energy density associated with the exchange interaction with exchange stiffness *J*_ex_, perpendicular magnetic anisotropy with anisotropy constant *K*_0_ and Dzyaloshinskii–Moriya interaction (DMI) with DMI constant *D*, respectively. The proposed AFM skyrmion-based diode was simulated by using a KMnF_3_ material that has excellent moisture resistance, a favorable bandgap of about 1.6 eV, and superb transport properties with 18 cm^2^ V^−1^ s^−1^ mobility.

This high mobility leads to a large spin diffusion length that reduces the deformation of the skyrmion in the presence of notches, thus enhancing the stability of a skyrmion in KMnF_3_ for long-term transportation.^37,38^ The material parameters used in the micromagnetic simulations were as follows:^28,39^ the nanotrack was considered to be 1024 × 300 × 1 nm^3^, cell size was 1 × 1 × 1 nm^3^. *M*_s,_*α*, *P*, and *J*_ex_ were 376 kA m^−1^, 0.005, 0.4, and −6.59 × 10^−12^ J m^−1^, respectively. Magnetic anisotropy and interfacial DMI were considered as 1.22 × 10^5^ J m^−3^ and 1.2 × 10^−3^ J m^−2^, respectively.

## 3 Operation of the proposed device

**Fig. 1:**
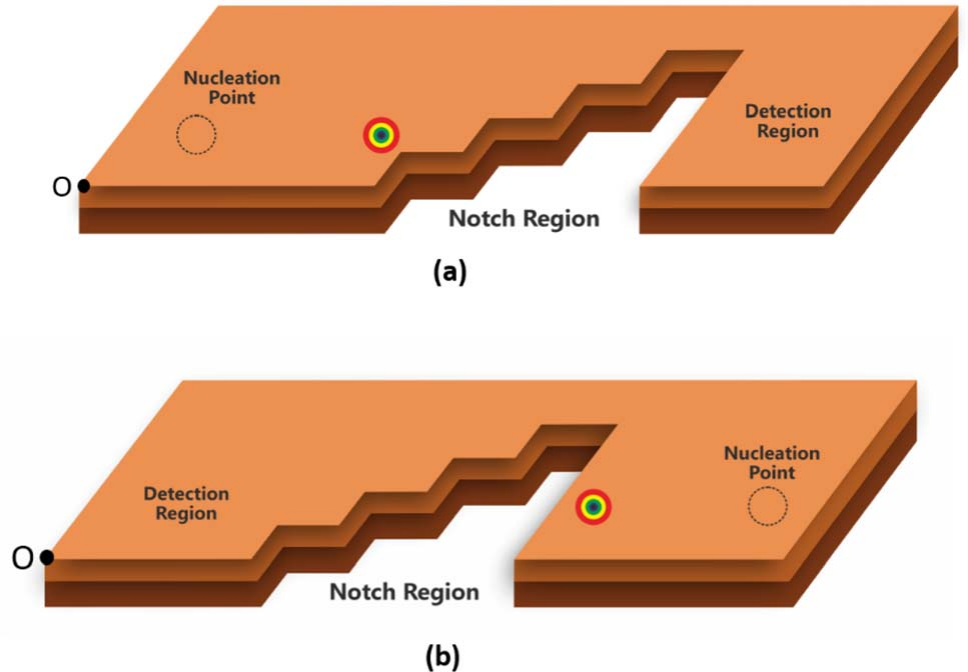
Schematic of the proposed device for (a) forward bias and (b) reverse bias.

**Fig. 2:**
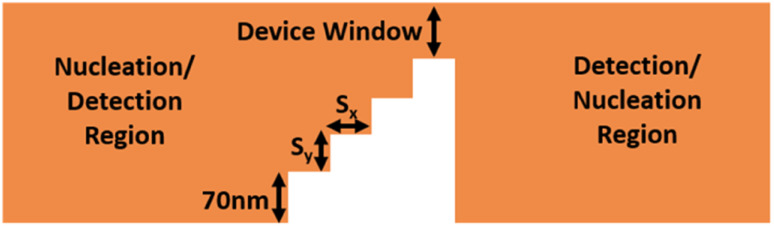
Attributes of the proposed device.

Fig. 1(a) and (b) show schematics of the proposed AFM skyrmion-based diode for both forward and reverse bias, respectively. This device has three primary components, namely the nucleation point, four-step notch region, and a detection region. In the case of forward and reverse bias, the AFM Neel skyrmion is initially created at the nucleation point *P*_0_ = (*x*_0_, *y*_0_) = (100, 60) nm and (924, 60) nm, respectively with respect to the origin (O). A staircase is created in the middle of the nanotrack to display the impact of repulsive forces from the step edges, thereby facilitating the one-way motion of the skyrmion. The skyrmion is observed in the detection region once it overcomes the device window. The whole nanotrack has a fixed length and width as 1024 nm and 300 nm, respectively. A detection region has a length of 400 nm. Fig. 2 illustrates the attributes of the proposed device. Here, *S*_*x*_ and *S*_*y*_ represent the horizontal and vertical step lengths of the notch region, respectively. The device window is the control gate for the skyrmion during the forward/reverse motion. The 1^st^ step vertical length is fixed as 70 nm irrespective of *S*_*x*_ and *S*_*y*_. This is considered in such a way that when the skyrmion is moving in the forward direction, it should directly reach the first step corner so that the skyrmion immediately starts experiencing the force in the +*y* direction that facilitates its motion towards the detection region.

**Fig. 3:**
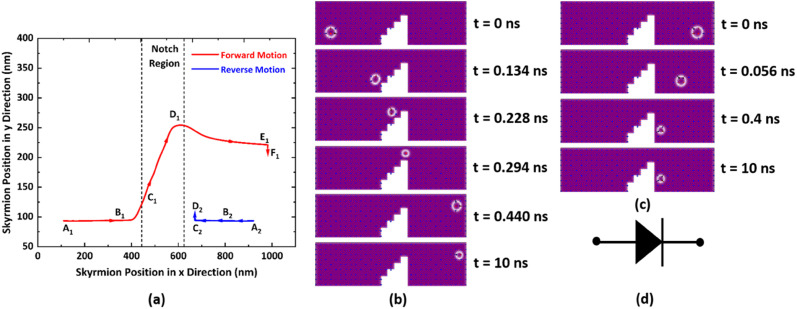
(a) Trajectories of the skyrmion for forward and reverse motion. (b and c) Snapshots of micromagnetic simulation at various positions for forward and reverse moving skyrmions, respectively. (Here, current density *J*, horizontal and vertical step lengths (i.e. *S_x_* and *S_y_*) are considered to be 20 GAm^−2^, 45 nm, 45 nm, respectively). (d) Diode symbol.

For the AFM skyrmion-based device to show diode functionality, it is mandatory to achieve a one-way motion of the skyrmion, apart from avoiding the deformation/annihilation of the skyrmion^12^ around the notch region. In the proposed device, a notch region facilitates the unidirectional motion of the skyrmion by bringing the significance of micromagnetic interaction energy that leads to repulsive forces on the skyrmion.^40^ In the case of forward motion, the skyrmion is nucleated at the left side (A1 point in Fig. 3) of the nanotrack, which is further drifted in the +*x* direction by the application of SOT. Once the skyrmion reaches near the notch region, the interaction between the core of the skyrmion and the notch edges enhance the potential energy. Hence, the spatial gradient 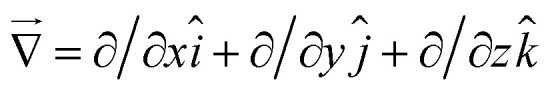 of the potential energy (*U*) gives rise to multiple repulsive forces (
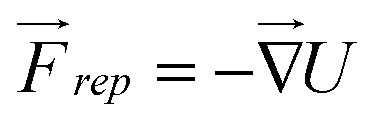
) from the vertical step, and from the horizontal step and the geometric edge in both the *x* and *y* directions, respectively, which can be represented as: *F⃑*_*x*_ = −∂*U*/∂*xî* and *F⃑*_*y*_ = −∂*U*/∂*yĵ*. These forces along with the force due to driving current (*F⃑*_total_ = *F⃑*_rep_ + *F⃑*_SOT_) will lead to change in the position of the skyrmion position with respect to time, *i.e. P*(*t*) = (*x*(*t*), *y*(*t*)). An analysis of the skyrmion motion due to various forces acting in both the transverse and longitudinal directions determine the skyrmion's ability to overcome the device window and reach the detection region. When the skyrmion reaches the other side, it can be detected through the magnetic tunnel junction (MTJ) reader according to the tunnelling magnetoresistance effect.^41^ If the force due to the driving current is not sufficient, the skyrmion will be pinned near the device window. Alternatively, for high current densities, there is a probability of skyrmion deformation/annihilation. Hence, the analysis of the driving current and the horizontal and vertical step lengths (*S*_*x*_ and *S*_*y*_) is required for proper device functionality, which is discussed in the next section.

In the case of reverse motion, the skyrmion is nucleated at the right side (A2 point in Fig. 3) of the nanotrack, which is driven in the −*x* direction using the driving current. The large vertical notch step (*i.e.* 205 nm as *S*_*y*_ = 45 nm) abruptly changes the potential energy, thereby acting as an energy barrier for the reverse-moving skyrmion. This generates a repulsive force in the direction opposite to the skyrmion motion. Hence, the skyrmion will not be able to overcome the device window, even for the case of high current densities, *i.e.* 40 GA m^−2^ (where 1 Giga Ampere (GA) = 10^9^ A). Consequently, only one-way motion of the skyrmion can be realized when a staircase notch is created in the midst of the nanotrack. Snapshots of micromagnetic simulations for both forward and reverse bias are shown in Fig. 3(b) and (c), respectively, along with the conventional diode symbol (Fig. 3(d)).

## 4 Results and discussion

### Working window of the proposed device

**Fig. 4:**
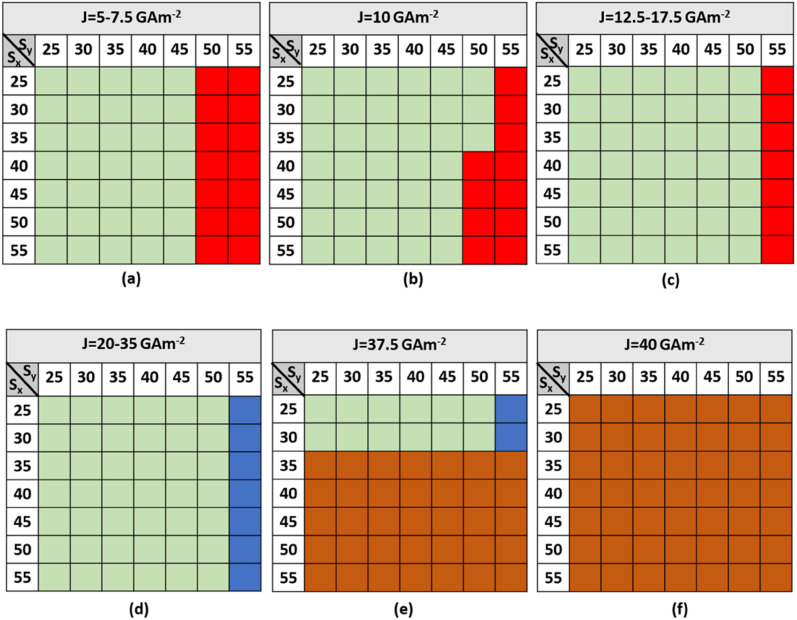
State diagrams for a forward-moving skyrmion with different horizontal and vertical step dimensions of the notch region for (a) 5 GA m^−2^ ≤ *J* ≤ 7.5 GA m^−2^ (b) *J* = 10 GA m^−2^ (c) 12.5 GA m^−2^ ≤ *J* ≤ 17.5 GA m^−2^ (d) 20 GA m^−2^ ≤ *J* ≤ 35 GA m^−2^ (e) *J* = 37.5 GA m^−2^ (f) *J* = 40 GA m^−2^.

**Fig. 5:**
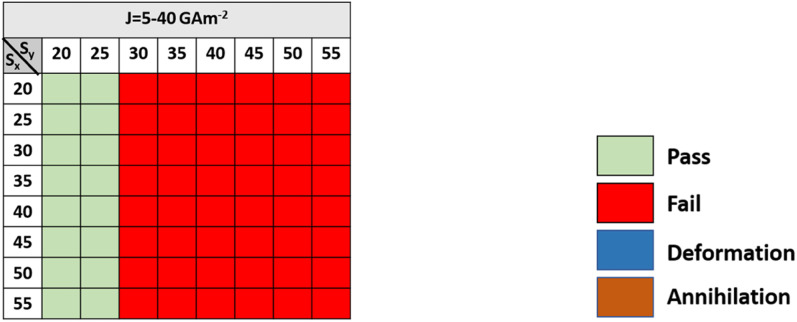
State diagram for a reverse-moving skyrmion with different horizontal and vertical step dimensions of the notch region for driving current densities in the range of 5–40 GA m^−2^.

The horizontal and vertical step dimensions of the notch region, as well as the driving current, have a huge impact on the performance of the AFM skyrmion-based diode. Fig. 4 and 5 show the state diagrams with various horizontal and vertical step dimensions for forward and reverse moving skyrmions, respectively, under different driving current densities (5 GA m^−2^ ≤ *J* ≤ 40 GA m^−2^). It is worth noting that for higher current densities (*J* ≥ 37.5 GA m^−2^), the skyrmions are annihilated even at smaller step dimensions. Moreover, the lower the current density, the more likely it is for the forward-moving skyrmion to reach the detection region without any deformation/annihilation.

From Fig. 5, it can be observed that a reverse-moving skyrmion can bypass the device window if *S*_*y*_ < 30. But, when *S*_*y*_ > 30 then due to the large single vertical step (*i.e.* ≥160 nm) it is not possible for the reverse-moving skyrmion to overcome the device window. Hence, it can be stated that there should always be an abrupt change in the large single vertical step to prevent the reverse moving skyrmion reach the detection region. Hence, to facilitate one-way functionality of the skyrmion, the driving current density, horizontal and vertical step dimensions should be considered in the ranges of 5 GA m^−2^ ≤ *J* ≤ 35 GA m^−2^, 25 nm ≤ *S*_*x*_ ≤ 55 nm, and 30 nm ≤ *S*_*y*_ ≤ 45 nm, respectively. The following are the four dynamical behaviors of the forward-moving skyrmion that are observed for different combinations of step dimensions and driving current densities:

#### Behavior 1: pass

It is worth noting from Fig. 4 that when 5 GA m^−2^ ≤ *J* ≤ 10 GA m^−2^, for 25 nm ≤ *S*_*x*_ ≤ 55 nm and 30 nm ≤ *S*_*y*_ ≤ 45 nm, and, when 12.5 GA m^−2^ ≤ *J* ≤ 35 GA m^−2^ for 30 nm ≤ *S*_*y*_ ≤ 50 nm, the skyrmion will bypass the device window. The driving current forces the skyrmion to move in the +*x* direction. As soon as it reaches near the notch region, repulsive forces in the −*x* and +*y* directions from the vertical and horizontal steps, respectively, also start acting on it. Hence, owing to the competition between all the forces in longitudinal and transverse directions, the skyrmion drifts diagonally towards the device window and reaches the detection region, as shown in Fig. 3(b).

#### Behavior 2: pinned

**Fig. 6:**
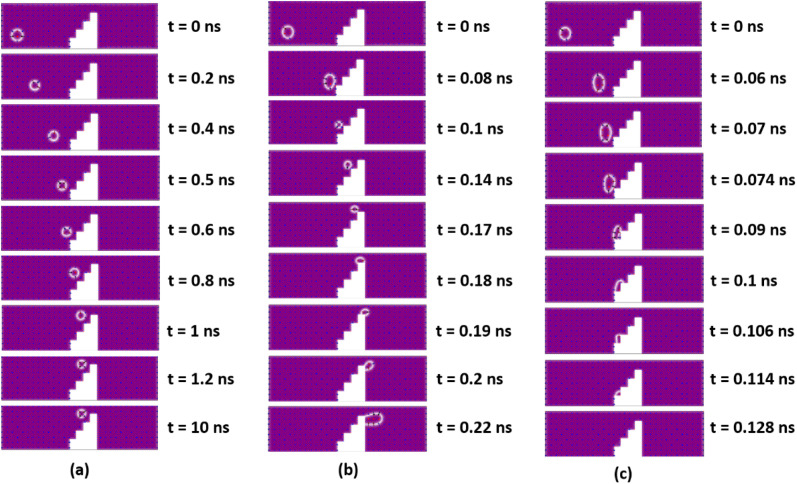
Micromagnetic simulations illustrating (a) pinned, (b) deform (c) annihilate behaviors of a forward-moving skyrmion.

For 50 nm ≤ *S*_*y*_ ≤ 55 nm with *J* = 5–10 GA m^−2^, the skyrmion will be pinned in between the third and fourth steps of the notch region, as shown in Fig. 6(a). Here, the potential energy gradient is steeper in the *x* compared to in the *y* direction. Hence, more repulsive force in the +*y* direction is required for the skyrmion to reach the detection region. When the skyrmion gradually reaches near to the fourth step, the force due to the geometric edge in the −*y* direction also comes into play. Due to the balancing of the forces in the +*x*, −*x*, +*y*, and -y directions, the skyrmion is pinned and will not be able to overcome the device window. However, with increase in current density to 12.5 GA m^−2^ ≤ *J* ≤ 35 GA m^−2^, the skyrmion will be able to reach the detection region, even for *S*_*y*_ = 50 nm, because the force due to current dominates over the other three repulsive forces acting on the skyrmion.

#### Behavior 3: deform (domain stripe)

For *S*_*y*_ = 55 nm with a current density of 20 GA m^−2^ ≤ *J* ≤ 35 GA m^−2^, when the skyrmion is about to cross the device window, the distance between the notch corner and the core of the skyrmion is almost negligible, which leads to high interaction between them. The skyrmion tries to attain annihilation in the fourth step due to the continuous repulsion force acting on the skyrmion in the −*y* direction from the geometrical edge. But, for a high current density range, skyrmion does not become annihilated, it just expands and deforms into the domain stripe, as shown in Fig. 6(b). This skyrmion behavior is also observed for some combinations of *S*_*x*_ and *S*_*y*_ at high current densities.

#### Behavior 4: annihilate

For 30 nm ≤ *S*_*y*_ ≤ 55 nm and 35 nm ≤ *S*_*x*_ ≤ 55 nm with a current density of *J* = 37.5 GA m^−2^, the working window is very narrow. Skyrmion annihilation occurs at the second or third steps of the notch region, as shown in Fig. 6(c). Owing to very high current density, the skyrmion is accelerated in the +*x* direction, which reduces the distance between the step corners and the skyrmion to a great extent. This leads to very high interaction of the skyrmion with the step corner, thereby, annihilating the skyrmion in the intermediate notch region. Moreover, with a current density of *J* = 40 GA m^−2^, the skyrmion is annihilated in the notch region irrespective of the step dimensions *S*_*x*_ and *S*_*y*_. The proposed device under the effect of temperature is discussed in ESI Note 1.†

### Impact of current density and notch with *S*_*x*_ = *S*_*y*_ on the skyrmion dynamics

**Fig. 7:**
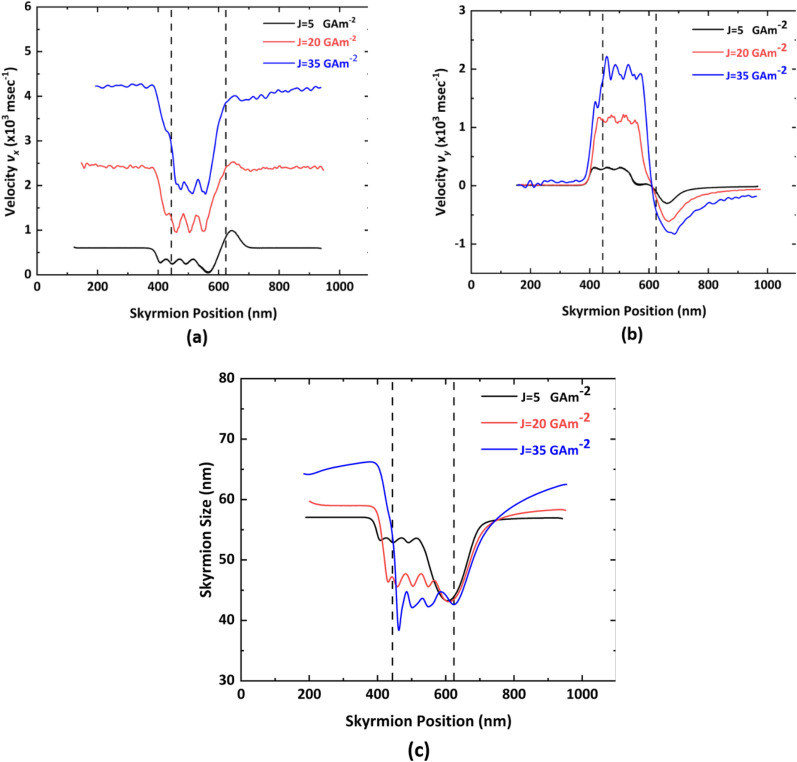
Variation of (a) velocity *v*_*x*_, (b) velocity *v*_*y*_, and (c) skyrmion size with respect to skyrmion position under different current densities for *S*_*x*_ = *S*_*y*_ = 45 nm in case of forward-moving skyrmion. Here, the notch region is between 444 nm and 624 nm represented by dotted lines.

**Fig. 8:**
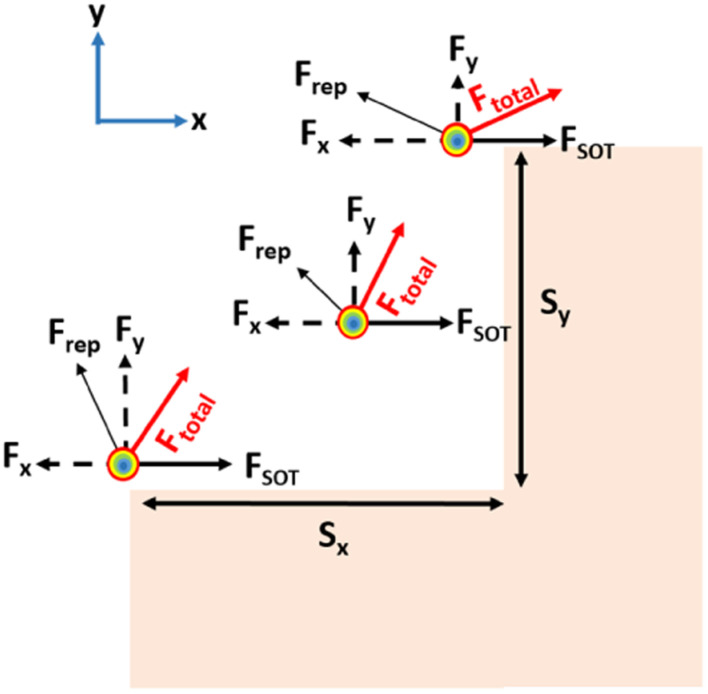
The total force (*F*_total_) acting on the skyrmion due to SOT (*F*_SOT_) and repulsion forces *F*_*x*_ and *F*_*y*_ from the vertical and horizontal steps, respectively, at different skyrmion positions.

The velocity of the skyrmion on a nanotrack with respect to the skyrmion position is shown in Fig. 7(a) and (b). However, the variation in the skyrmion size with respect to its position is illustrated in Fig. 7(c). This is obtained for *S*_*x*_ = *S*_*y*_ = 45 nm under three different current densities *i.e. J* = 5GA m^−2^, *J* = 20 GA m^−2^, and *J* = 35 GA m^−2^. At first, the skyrmion follows a straight trajectory until it reaches the notch region. The *x* component of the velocity (*v*_*x*_) is directly proportional to the driving current density *J*.^42^ In this particular case, there is an approximately 300% increase in *v*_*x*_ with a 300% increase in the current density whenever there is a negligible interaction between the skyrmion and the notch region. Moreover, the *y* component of the velocity (*v*_*y*_) before the notch region is zero due to the fact that an AFM skyrmion does not exhibit SkHE. Once the skyrmion reaches near the first step of the notch region, the skyrmion starts experiencing repulsion force from the vertical and horizontal steps, thereby deaccelerating in the −*x* direction against the driving force due to SOT and accelerating in the +*y* direction at that moment. Thereafter, the oscillatory behavior of the skyrmion is visible in both the velocity components *v*_*x*_ and *v*_*y*_ in the notch region. This is due to the fact that the distance between the core of the skyrmion and the vertical/horizontal step is varying continuously while the skyrmion is moving diagonally towards the device window. The maxima and minima of the oscillations are observed when the skyrmion is just before or after the step corner. This phenomenon is illustrated in Fig. 8. When the skyrmion reaches the device window, it will experience negligible repulsive force in the *y* direction. Hence, the driving current force accelerates the skyrmion in the +*x* direction. Once the skyrmion crosses the device window, it attains the uniform velocity *v*_*x*_ but the force due to the geometric edge will act on the skyrmion in the −*y* direction, thereby moving the skyrmion in downward direction to some extent. After a few seconds, there will not be any movement of the skyrmion in the *y* direction due to an increase in the distance between the skyrmion and the geometric edge in the detection region. It is also observed that the processing speed of the proposed device is in the range of 600–4200 ms^−1^ for current densities of *J* = 5–35 GA m^−2^. A comparison of the numerical and analytical results of the AFM skyrmion velocity in the case of negligible impact of the repulsion force on the skyrmion from the notch edges is presented in the ESI Note 2.†

The skyrmion size is strongly affected by the total energy, which is the sum of the exchange energy, anisotropy energy, energy due to driving current, and interaction from the edges.^43^ For the proposed device, the change in the size of the skyrmion with respect to its position is shown in Fig. 7(c). For a specific current density, the size of the skyrmion will remain almost constant unless and until it reaches the notch region owing to the negligible interaction between the skyrmion and the notch edges. Once, the skyrmion reaches near the notch region, the size is reduced to some extent, and furthermore, it starts exhibiting oscillatory behavior while moving towards the device window by virtue of the different repulsion forces acting at the various steps. It can thus be concluded that the higher the repulsion force, the lower the skyrmion size.^12^ In the device window, the skyrmion size will be at a minimum as there will be an additional repulsion force at this moment. Once it overcomes the device window, it regains its initial size. Moreover, with an increase in the driving current density, the size of the AFM skyrmion is enlarged.^44^ Under large current densities, the skyrmion experiences very high repulsive force by virtue of increased potential energy gradient. Hence, the reduction in the skyrmion size is at a maximum. Alternatively, the amount of reduction in size is less for low current densities.

**Fig. 9:**
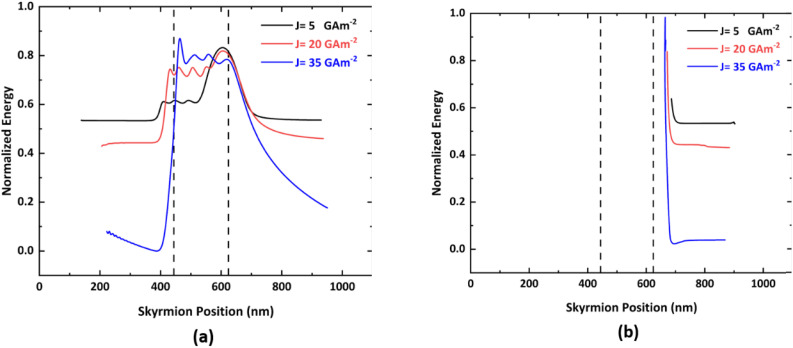
Variation of the total energy of the skyrmion with respect to skyrmion position under different current densities for *S*_*x*_ = *S*_*y*_ = 45 nm in the case of (a) forward-moving skyrmion and (b) reverse-moving skyrmion.

The variation in the normalized energy of a skyrmion with respect to the skyrmion position is shown in Fig. 9. During the forward motion of the skyrmion, the potential energy exhibits several sharp changes. These changes correspond to the different steps of the notch region, as when the skyrmion approaches the ends of all four steps of the notch region, oscillatory behavior is observed by virtue of the varying interaction between the skyrmion and the notch edge.

With an increase in current, the distance between the skyrmion and the notch edges decreases significantly, thereby leading to an increase in the skyrmion boundary interaction potential.^45^ During the reverse motion of the skyrmion, there is a large single vertical step that will abruptly increase the potential energy gradient, thereby exerting a huge amount of repulsive force on the skyrmion and preventing two-way motion of the skyrmion. In addition, assuming the width and thickness of heavy metal (HM) to be 300 nm and 2 nm, respectively, and an input current density of 20 GA m^−2^, the average current flowing through the HM is *I*_HM_ = 12 μA. The process time (*t*_p_) is 0.4 ns and the resistivity of the HM (*ρ*_HM_) is assumed to be 1800 nΩ m.^18^ Consequently, the estimated total energy dissipation is 0.176 fJ. These advantages of the proposed device could further minimize the energy consumption of future AFM skyrmionic devices for information processing. Moreover, the comparison of the proposed device with other skyrmion based diodes is included in ESI Note 3.†

### Impact of current density and asymmetric (*S*_*x*_ ≠ *S*_*y*_) notch region on the skyrmion dynamics

**Fig. 10:**
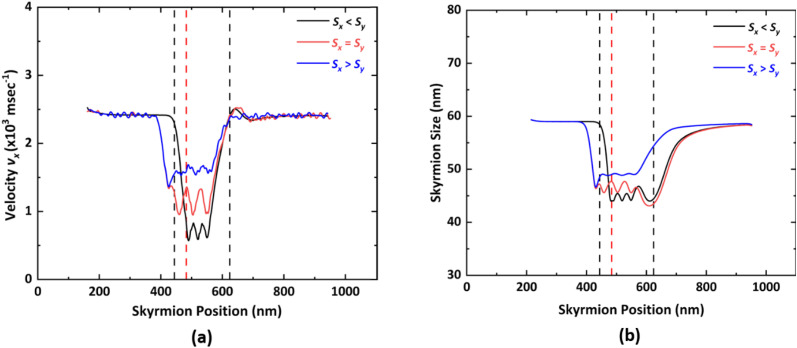
Variation of (a) velocity *v*_*x*_ and (b) skyrmion size with respect to skyrmion position under different step ratios for current density *J* = 20 GA m^−2^ in case of forward-moving skyrmion. For *S*_*x*_ = *S*_*y*_ and *S*_*x*_ > *S*_*y*_, notch region is between 444 nm to 624 nm. For *S*_*x*_ < *S*_*y*_, notch region is between 484 nm and 624 nm, represented by dotted lines.

While fabricating the proposed device, the step dimensions *S*_*x*_ and *S*_*y*_ might not be exactly equal, which makes the analysis of impact of the driving current density and notch with *S*_*x*_ ≠ *S*_*y*_ on skyrmion dynamics an important aspect. Fig. 10(a) and (b) show the variation in the velocity *v*_*x*_ and skyrmion size with respect to the skyrmion position, respectively for different step ratios, *i.e. S*_*x*_ < *S*_*y*_, *S*_*x*_ = *S*_*y*_ and *S*_*x*_ > *S*_*y*_. When *S*_*x*_ < *S*_*y*_, the repulsion from the vertical step in the −*x* direction dominates over the repulsion from the horizontal step in the +*y* direction, which reduces the velocity *v*_*x*_ as well as the skyrmion size to the highest extent near the notch region. Moreover, if *S*_*x*_ > *S*_*y*_, then the repulsion in the +*y* direction dominates over repulsion in the −*x* direction. Hence, for *S*_*x*_ > *S*_*y*_, the velocity *v*_*x*_ and the skyrmion size are much higher compared to that for *S*_*x*_ < *S*_*y*_.

## Conclusion

An AFM skyrmion-based diode was designed using a staircase notch region at the middle of the nanotrack, which makes the proposed device highly energy efficient. The notch region was exploited to induce a change in potential energy during the skyrmion motion, which led to various repulsive forces acting on it. The physical realization of a notch in any device is a challenging task. However, it is possible to achieve the precise notch dimensions using high-quality fabrication techniques.^46–48^ In this work, the skyrmion motion under different driving current densities and horizontal and vertical step dimensions has been demonstrated to identify the proper working window of the proposed device. The research results show that to achieve one-way motion of the skyrmion, *i.e.* motion under different driving current densities and horizontal and vertical step dimensions has been demonstrated to identify the diode functionality, the device must be operated under in the range of 5 GA m^−2^ ≤ *J* ≤ 35 GA m^−2^, 25 nm ≤ *S*_*x*_ ≤ 55 nm, and 30 nm ≤ *S*_*y*_ ≤ 45 nm. The forward-moving skyrmion will bypass the device window, while the reverse-moving skyrmion gets stuck near the large single vertical step. Here, we not only analyzed the energy variation of the skyrmion during its motion but also identified the variation in the velocity and skyrmion size during the skyrmion motion on the nanotrack under different driving current densities and geometric parameters of the notch region. This device offers very low energy consumption and high processing speed, making it a potential candidate for the implementation of future AFM skyrmionic devices for information processing.

## Conflicts of interest

There are no conflicts to declare.

## Supplementary Material

NA-005-D2NA00748G-s001
