# Supplementary material for: Antiferromagnetic skyrmion-based high speed diode
Source: Nanoscale Adv. 2022 Dec 15;5(2):450–8. doi: 10.1039/d2na00748g (PMC9846466; doi:10.1039/d2na00748g)
Supplement: NA-005-D2NA00748G-s001 [file NA-005-D2NA00748G-s001.pdf]

Electronic Supplementary Material (ESI) for Nanoscale Advances.
This journal is © The Royal Society of Chemistry 2022

Antiferromagnetic Skyrmion based High Speed Diode

Namita Bindal,*[a] Ravish Kumar Raj [b], and Brajesh Kumar Kaushik [c]

[a]namita@ec.iitr.ac.in, [b]ravish_kr@ece.iitr.ac.in, [c]bkk23fec@iitr.ac.in

Supplementary Information

Supplementary Note 1: The effect of temperature on the proposed device

The effect of the finite temperature (i.e. 50K, 100K, and 150K) on the operation of AFM skyrmion based diode is studied for $S_x = S_y = 45nm$ at current density $J = 5 \times 10^9 Am^{-2}$. As shown in Figure S1, under forward bias, at T = 0 K, the skyrmion is able to reach the detection region. As T increases to 100K, under thermal fluctuations, the skyrmion will still be able to reach the detection region with more propagation velocity due to more energy being generated under thermal fluctuations. However, at T=150K, the skyrmion gets annihilated at the fourth step. This is due to the fact that with rise in temperature, the degree of deformation of the skyrmion increases.

Case 1 Forward Bias:

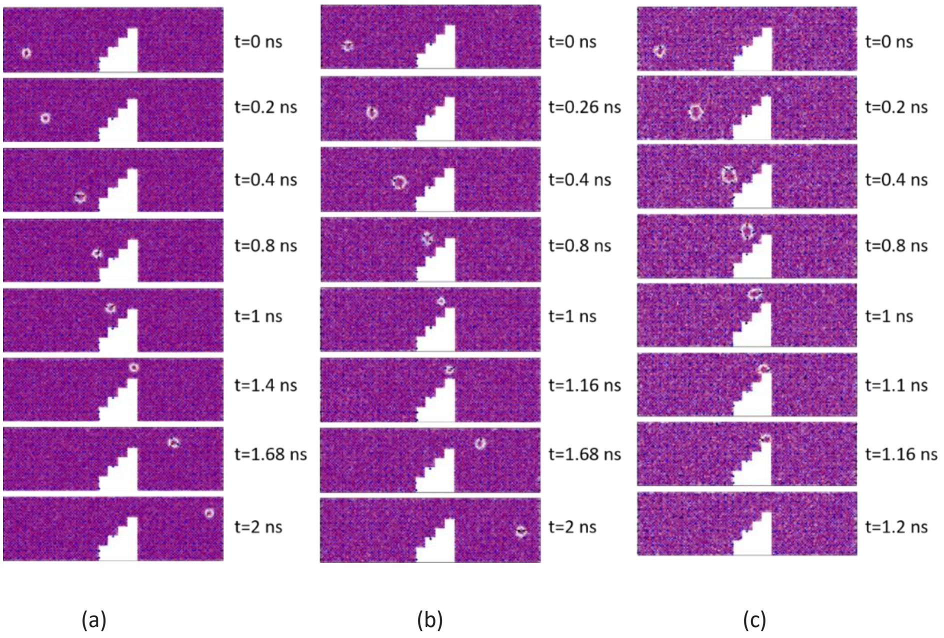

Figure S1: Micromagnetic simulations for $S_x = S_y = 45nm$, current density $J = 5 \times 10^9 Am^{-2}$ and temperature a) 50K b) 100K c) 150K for forward-moving skyrmion.

Figure S2 depicts that under reverse bias, within the temperature range 0-150K, the large vertical notch step abruptly changes the potential energy and generates the high repulsive force in the direction opposite to the skyrmion motion. Hence, no reverse motion of skyrmion is seen. Thus, within the temperature range 0-100K, the proposed device facilitates the one-way motion of the skyrmion for $S_x = S_y = 45nm$ at current density of $J = 5 \times 10^9 Am^{-2}$.

Case 2 Reverse Bias:

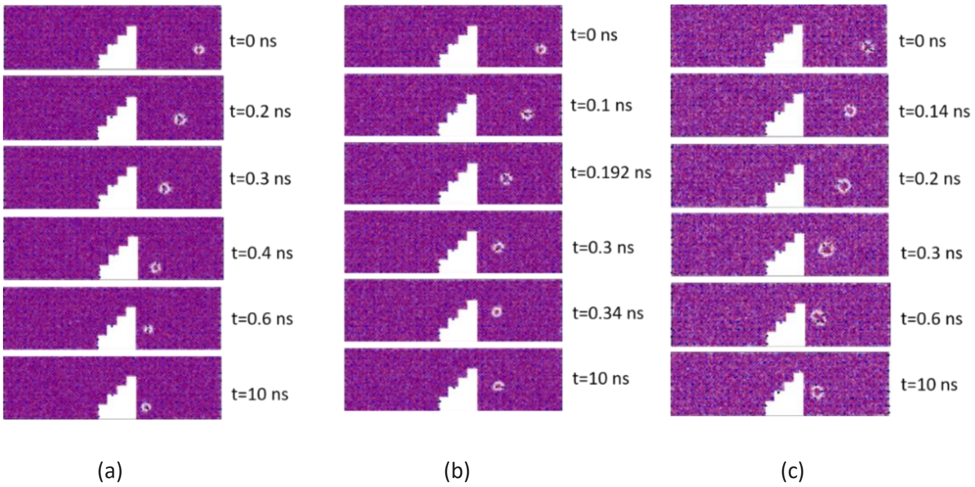

Figure S2: Micromagnetic simulations for $S_x = S_y = 45nm$, current density $J = 5 \times 10^9 Am^{-2}$ and temperature a) 50K b) 100K c) 150K for reverse-moving skyrmion.

Supplementary Note 2: Comparison of numerical and analytical results of the skyrmion velocity

The Thiele equation for the skyrmion is described as follows [S1-S4]:

$$\vec{G} \times \vec{v} - \alpha\overleftrightarrow{D}.\vec{v} + \vec{F}_{total} = 0 \tag{1.1}$$

Considering the topology of the AFM skyrmion that doesn't exhibit SkHE, the first term of equation 1.1 i.e. Magnus force is zero and equation 1.1. is re-written as follows:

$$0 - \alpha\overleftrightarrow{D}.\vec{v} + \vec{F}_{SOT} + \vec{F}_{rep} = 0 \tag{1.2}$$

$$-\alpha\overleftrightarrow{D}.\vec{v} + \vec{F}_{SOT} + \vec{F}_x + \vec{F}_y = 0 \tag{1.3}$$

where, α is the damping constant and *v* is the AFM skyrmion velocity. *D* is the dissipative tensor that is given as $\overleftrightarrow{D} = \begin{pmatrix} D & 0 \\ 0 & D \end{pmatrix}$ with $D = \int dxdy\delta_x\vec{m}.\delta_x\vec{m} = \int dxdy\delta_y\vec{m}.\delta_y\vec{m}$ [S5-S7]. However, F_{SOT} represents the force due to spin orbit torque (SOT) that is defined as follows [S5]:

$$F_{SOT} \approx \gamma H_0 I \tag{1.4}$$

where, γ is the gyromagnetic ratio and $H_0 = (\hbar J\theta_{SHE})/(2e\mu_0 t_0 M_s)$. Here, $\hbar$, J, θ_{SHE}, e, μ_0, t_0, and M_s are the reduced Planck's constant, charge current, spin hall angle, electron charge, absolute permeability of free space, thickness of the film, and saturation magnetization, respectively. $I \approx \pi^2 R_s$ where R_s is the radius of the skyrmion [S4,S6,S7]. However, $\vec{F}_x = -\frac{\partial U}{\partial x}$ is the repulsion force acting on the skyrmion in *x* direction due to the vertical step whereas $\vec{F}_y = -\frac{\partial U}{\partial y}$ is the repulsion force acting on the skyrmion in *y* direction due to the horizontal step of the notch region. By solving equations 1.3 and 1.4, the speed of the AFM skyrmion in its components is defined as follows:

$$v_x + v_y = \frac{F_{SOT} + F_x + F_y}{\alpha D} \tag{1.5}$$

$$v_x = \frac{(F_{SOT} + F_x)}{\alpha D} \tag{1.6}$$

$$v_y = \frac{F_y}{\alpha D} \tag{1.7}$$

Whenever the skyrmion is at much larger distance from the notch region, there will be negligible impact of the repulsion force from the notch edges. i.e. $F_x = F_y = 0$. Hence, the speed of the AFM skyrmion in its components is defined as follows:

$$v_x = \frac{F_{SOT}}{\alpha D} \text{ and } v_y = 0 \tag{1.8}$$

The AFM skyrmion speed at various current densities is illustrated in Figure S3.

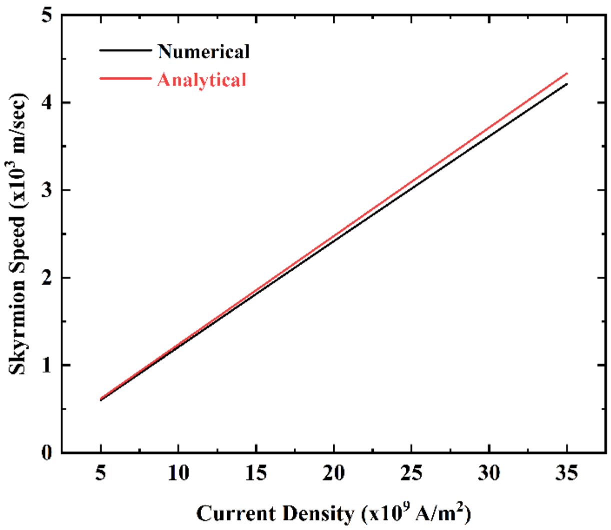

Figure S3: The simulation results and analytical results of the velocity of the skyrmion.

Supplementary Note 3: Comparison of the proposed device with other related works

Table S1 shows the comparison of skyrmion based diodes in terms of type of the skyrmion, racetrack materials, operating current densities, maximum average speed, and major functionalities. It concludes that the current density used in our work is much lesser than those used in Refs [S8-S11]. Also, the average speed attained in our proposed device is much larger as compared to other diode devices even when the operating current is small. Hence, our proposed device opens up the path for the development of high-speed devices in antiferromagnetic spintronics.

Table S1 Comparison of our design with other related works

**Skyrmion Type**	**Current Density ($\times 10^{10}$ A/m^{-2})**	**Maximum Average Speed (m/sec)**	**Device Design**
FM skyrmion [S8-S11]	2-60	30-120	✓ Various methods have been employed to control the skyrmion transport such as high anisotropy regions, assymetric structures, modified edges and skyrmion hall effect.
**AFM skyrmion (This work)**	**0.5**	**600**	✓ **The skyrmion transport is controlled by exploiting the staircase notch region in the middle of the nanotrack.**

References

[S1] Y. Ishida and K. Kondo, *Journal of Magnetism and Magnetic Materials*, 2020, **493**, 165687.

[S2] L. Shen *et al.*, *Applied Physics Letters*, 2019, **114**, 042402.

[S3] C. C. I. Ang, W. Gan, and W. S. Lew, *New Journal of Physics*, 2019, **21**, 043006.

[S4] Y. Shu *et al.*, *Applied Physics Letters*, 2022, **121**, 042402.

[S5] L. Shen *et al.*, *Physical Review Letters*, 2020, 124, 037202.

[S6] R. M. Menezes, J. Mulkers, C. C. S. Silva, and M. V. Miloševic, *Physical Review B*, 2019, **99**, 104409.

[S7] L. Shen *et al.*, *Physical Review B*, 2018, **98**, 134448.

[S8] L. Zhao *et al.*, *Nanoscale*, 2020, **12**, 9507.

[S9] D. H. Jung *et al.*, *Physical Review B*, 2021, **104**, L060408.

[S10] Y. Shu *et al.*, *Applied Physics Letters*, 2022, **121**, 042402.

[S11] Y. Feng *et al.*, *IEEE Transactions on Electron Devices*, 2022, **69**, 3.
